# PdRu bimetallic nanoalloys with improved photothermal effect for amplified ROS-mediated tumor therapy

**DOI:** 10.3389/fbioe.2024.1523599

**Published:** 2025-01-03

**Authors:** Yujia Liang, Shufang Ning, Mekhrdod S. Kurboniyon, Khaiyom Rahmonov, Zhengmin Cai, Shirong Li, Jinling Mai, Xiaojing He, Lijuan Liu, Liping Tang, Litu Zhang, Chen Wang

**Affiliations:** ^1^ Department of Experimental Research and Guangxi Cancer Molecular Medicine Engineering Research Center and Guangxi Key Laboratory of Basic and Translational Research for Colorectal Cancer, Guangxi Medical University Cancer Hospital, Nanning, China; ^2^ National Academy of Sciences of Tajikistan, Dushanbe, Tajikistan; ^3^ Department of Information, Library of Guangxi Medical University, Nanning, China

**Keywords:** nanoalloy, reactive oxygen species, glutathione, photothermal effect, tumor therapy

## Abstract

An emerging strategy in cancer therapy involves inducing reactive oxygen species (ROS), specifically within tumors using nanozymes. However, existing nanozymes suffer from limitations such as low reactivity, poor biocompatibility, and limited targeting capabilities, hindering their therapeutic efficacy. In response, the PdRu@PEI bimetallic nanoalloys were constructed with well-catalytic activities and effective separation of charges, which can catalyze hydrogen peroxide (H_2_O_2_) to toxic hydroxyl radical (·OH) under near-infrared laser stimulation. Through facilitating electron transfer and enhancing active sites, the enhanced peroxidase-like (POD-like) enzymatic activity and glutathione (GSH) depletion abilities of nanozymes are boosted through a simple co-reduction process, leading to promising anti-tumor activity. The electron transfer between Pd and Ru of PdRu@PEI nanoalloys contributes to POD-like activity. Then, by oxidizing endogenous overexpressed GSH, enzymatic cycling prevents GSH from consuming ROS. Furthermore, the surface plasmon resonance effect of near-infrared laser on bimetallic nanoalloys ensures its photothermal performance and its local heating, further promoting POD-like activity. The integrated multi-modal therapeutic approach of PdRu@PEI has demonstrated significant anti-cancer effects *in vivo* studies. The nanozymes exhibit high catalytic efficiency and excellent biocompatibility, offering valuable insights for the development of nano-catalysts/enzymes for biomedical applications.

## 1 Introduction

With increasing global morbidity and mortality rates, cancer remains a serious threat to human health (Zhou et al., 2024; Zhao P. Et Al., 2023; Diao and Liu, 2023; Ai et al., 2023). Conventional treatment modalities, such as chemotherapy, radiotherapy, and surgical resection, struggle to achieve specific effects on malignant cells. As a result, these treatments inevitably cause irreversible damage to normal cells and tissues in the body, leading to poor patient prognosis. With the cross-vergence of nanomedicine and nano-catalysis, the utilization of nanozymes to trigger enzyme-catalyzed reactions under the unique physiological characteristics of the tumor microenvironment (TME) and achieving catalytic therapy with substrate specificity and low toxic side effects have become a novel strategy for tumor treatment (Zhang W. et al., 2024; Zhu et al., 2023a; Zhao Q. et al., 2023; Rong et al., 2023; Zhang R. et al., 2024). However, the harsh TME frequently compromises the catalytic activity of nanozymes under physiological conditions, thereby inhibiting the efficacy of cancer treatment, which motivates research toward modulating the TME and developing multi-modal synergistic therapies to amplify tumor suppression (Zandieh and Liu, 2021; Yu et al., 2021; Wang et al., 2021; Xiaoli et al., 2021; Duan et al., 2023; Zhu et al., 2023b). TME is the unique microenvironment of tumor cells and their surroundings relating to tumor formation and metastasis (Zeng et al., 2023; Yang Y. et al., 2023a; Yang X. et al., 2023b; Yang L. et al., 2023c). TME includes not only peripheral micro-vessels, fibroblasts, lymphocytes, immune cells, bone marrow-derived inflammatory cell signaling molecules, and other extracellular matrices but also varieties of biological features within tumor tissues, such as O_2_ content, pH, and redox environment (Wu et al., 2023; Wang H. et al., 2023; Tao et al., 2023; Song et al., 2023; Shi et al., 2023). Using abnormal biochemical markers in the tumor tissue microenvironment and tumor cells is a potential target for exploring and developing new therapeutic modalities. Reactive oxygen species (ROS) are a series of molecules with strong oxidizing capacity, which is a by-product of aerobic metabolism and a special cell signaling transcription factor. ROS in living organisms mainly includes superoxide radicals (O_2_^·−^), singlet oxygen (^1^O_2_), hydroxyl radicals (·OH), nitric oxide (NO), hydrogen peroxide (H_2_O_2_), et al. (Zhang H. et al., 2024; Xu et al., 2024; Chen et al., 2024; Zhang W. et al., 2023; Zhang Q. et al., 2023). ROS is not only implicated in protein folding, cell proliferation, differentiation and migration, and signaling but also implicated in respiration, defense responses, and oxidative damage. In biological cells, H_2_O_2_ is mainly derived from the single-electron reduction of oxygen molecules, first to form O_2_^·−^, and then catalyzed by superoxide dismutase (SOD) to form H_2_O_2_. H_2_O_2_ has a variety of catalytic activities and has been widely used as a raw material for the activation of drug release and the manufacture of intracellular oxygen (Wang S. et al., 2023; Wang R. et al., 2023; Wang D. et al., 2023; Pan et al., 2023). Tumor cells produce higher concentrations of H_2_O_2_ compared to normal cells. H_2_O_2_ can induce DNA damage and mutations, and increased H_2_O_2_ concentrations also activate hypoxia-inducible factor-1. H_2_O_2_ has high biological toxicity, largely due to its readiness to be converted into various types of reactive free radicals, disturbing the intracellular redox metabolic balance, especially by interacting with transition metal ions to generate highly reactive ·OH (Rong et al., 2023; Jiang et al., 2023; Huang et al., 2019).

In this study, we initially designed the bimetallic nanoalloys catalyst through a simple co-reduction method, enabling collaborative bimetallic peroxidase (POD)-like activity and surface plasmon resonance (SPR)-induced photothermal conversion (Wen et al., 2023; Nie et al., 2023; Liao et al., 2023). Herein, PdRu@PEI bimetallic nanoalloys exhibit specific enzymatic activity in generating toxic ROS through interaction with H_2_O_2_ in the acidic TME. Firstly, the electron transfer between Pd and Ru results in a reduction in the electron binding energy of Pd, facilitating the transfer of electrons from Pd to H_2_O_2_ and consequent ROS production. Secondly, the PdRu can consume glutathione (GSH), disrupting the antioxidant system balance and escalating oxidative stress. Meanwhile, PdRu@PEI nanoalloys exhibit photothermal effects under light irradiation, resulting in temperature changes conducive to enhancing the catalytic activity of PdRu@PEI bimetallic nanoalloys. In tumor treatment, this strategy, which combines the activity of POD-like enzymes using precious metal alloys as catalysts and the SPR effect of nanoalloys, holds significant promise for ROS-induced tumor treatment.

## 2 Experimental section

### 2.1 Chemicals and reagents

Oleic acid (OA, 90%), oleylamine (OAm, 98%), ruthenium chloride (RuCl_3_, 99%), carbonyl tungsten (W(CO)_6_, 98%), acetylacetone palladium (Pd (acac)_2_, 98%), polyethyleneimine (PEI, 99%, Mw25000), 1,3-diphenylisobenzofuran (DPBF, 97%), 3,3′,5,5′-tetramethyl-benzidine (TMB), and 5,5 dimethyl-1pyrroline N-oxide (DMPO) were purchased from Sigma-Aldrich. Methyl thiazolyl tetrazolium (MTT, >98%), fluorescein isothiocyanate (FITC, 95%), 4′,6-diamidino-2-phenylindole (DAPI), Calcein-AM (≥97%), propidium iodide (PI, ≥99%), JC-1 staining kit, and annexin V-FITC/PI apoptosis detection kit were obtained from Beyotime Inst. Biotech. The PBS and RPMI 1640 media were purchased from Gibco Life Technologies.

### 2.2 Characterization

Transmission electron microscopy (TEM) images were utilized by an FEI Tecnai T20 instrument. X-ray diffraction (XRD, RigakuD/MAX-TTR-III) measured the crystal structure of materials. Further, the ultraviolet-visible (UV-vis) absorbance spectra were conducted by a UV-1601 spectrophotometer. X-ray photoelectron spectroscopy (XPS, ESCALAB 250Xi) was used to analyze the valence of materials. The ·OH was detected by electron spin resonance (ESR, Bruker EMX1598). The inductively coupled plasma-mass spectrometer (ICP-MS) was operated on an Icap 6300.

### 2.3 Synthesis of PdRu@PEI nanoalloys

The Pd (acac)_2_ (20 mg), RuCl_3_ (14 mg), OAm (16 mL), andOA (4 mL) were sonicated for 10 min, heated to 110°C, and stirred for 20 min. The mixture was then heated to 240°C for another 40 min to reduce it to nucleation. The entire process was carried out under a nitrogen atmosphere. Centrifuge the PdRu bimetallic nanoalloys at 6,000 rpm and wash them with ethanol, cyclohexane, and 3 times. Preserve the product with cyclohexane. Dissolve the product in 50 mL of water, add 1 mL of PEI, stir at room temperature for 4 h, centrifuge and wash at 6000 rpm, and preserve the product PdRu@PEI with H_2_O.

### 2.4 ROS production activity estimation

After different treatments (808 nm irradiation, 50°C water bath, and control), PdRu@PEI (200 μg mL^–1^) was mixed with TMB (6 mg mL^−1^), H_2_O_2_, and PBS to evaluate the ROS production ability. Then, different concentrations of PdRu@PEI solutions were switched for further tests. Different treatments (808 nm irradiation, 50°C water bath, and control) were added to evaluate the POD-like activity. To test the enzymatic kinetic parameters, the UV absorption of the reaction system at 652 nm was measured by changing the concentration of H_2_O_2_ over time. The type of ROS was determined by mixing the DMPO, PdRu@PEI, and H_2_O_2_ and testing its ESR characteristic peaks.

### 2.5 GSH depletion estimation

After different treatments (808 nm irradiation, 50°C water bath, and control), PdRu@PEI (200 μg mL^–1^) was mixed with GSH (5 mM) and H_2_O_2_ (12.5 mM) in PBS solution. Followed by adding DTNB at 2, 4, 6, 8, and 10 min with a final concentration of 0.3 × 10^−3^ M.

### 2.6 Photothermal effect estimation

PdRu@PEI (25, 50, 100, and 200 μgmL^–1^) and pure water (control) were illuminated at room temperature for 300 s with an 808 nm laser (0.8 W cm^–2^). Then, PdRu@PEI (100 μgmL^–1^) was irradiated with 808 nm laser (0.4, 0.6, 0.8, and 1.0 W cm^–2^) for 300 s, and take photos using an infrared thermal imager (FLIR System E40). For four cycles, PdRu@PEI (100 μgmL^–1^) was irradiated at 0.8 W cm^–2^. Meanwhile, the PdRu@PEI (200 μgmL^–1^) was injected into the mouse tumor *in situ*, and the tumor was irradiated with an 808 nm laser (0.8 W cm^–2^) and photographed using an infrared thermal imager.

### 2.7 *In vitro* evaluation of cellular uptake

The CT26 (mouse colon cancer cells) was acquired from the Cell Bank. CT26 cells were put into the culture dish and grown for 24 hat 37°C in a humidified environment with 5% CO_2_. The cell culture media was changed with a new medium containing FITC-labeled PdRu@PEI (100 µgmL^–1^) for 0.5, 1, and 3 h, respectively, and their nuclei were stained with DAPI (10 μgmL^–1^) for 5 min at 37°C. The treated CT26 cells were washed twice with PBS and observed under a confocal laser scanning microscope (CLSM).

### 2.8 *In vitro* cell viability assay

The CT26 cells were seeded into 96-well plates and cultured for 24 h. Then, the cell culture medium was refreshed with a fresh culture medium containing PdRu@PEI with different concentrations. The light-related groups were irradiated with an 808 nm laser (0.8 W cm^−2^). CT26 cells were seeded and cultured for 24 h, then treated with the following conditions: 1. Control; 2. NIR (0.8 W cm^−2^, 3 min); 3. PdRu@PEI (100 µgmL^–1^); 4. PdRu@PEI (100 µgmL^–1^) + NIR (3 min). After co-culturing with samples for 24 h, followed by the addition of MTT (20 μL, 5 mgmL^–1^) and cultured for another 4 h. Finally, 150 μL of DMSO was added, and the absorbance was measured using Biotek Gen5. Additionally, the *in vitro* viability of PdRu@PEI to L929 fibroblast cells was evaluated using the same method.

### 2.9 Detection of intracellular ROS

The CT26 cells were seeded into 6-well plates (10^5^ per well) and incubated overnight for adherence. The cells were incubated for 4 h under five conditions: 1. Control; 2. NIR (0.8 W cm^−2^, 3 min); 3. PdRu@PEI (100 µgmL^−1^); 4. PdRu@PEI (100 µgmL^−1^) + NIR (3 min). Then, the cells were stained by DCFH-DA and DAPI for 20 min and observed by CLSM.

### 2.10 *In vitro* anticancer efficacy

The CT26 cells were treated with the following conditions: 1. Control; 2. NIR (0.8 W cm^−2^, 3 min); 3. PdRu@PEI (100 µgmL^−1^); 4. PdRu@PEI (100 µgmL^−1^) + NIR (3 min). After co-incubating at 37°C within 4 h, the NIR-related group was exposed to laser irradiation, and then the cells were co-incubated with Calcein AM and PI for 30 min. The CT26 cells were washed repeatedly with PBS and then imaged using CLSM. Then, the CT26 cells were double stained with the Annexin V-FITC Apoptosis Detection Kit, followed by the flow cytometric analysis.

### 2.11 *In vivo* anti-tumor estimation

The CT26 tumor-bearingBALB/c mice were intravenously administrated with PdRu@PEI (10 mgkg^−1^) at different times. The tumor and major organs were collected from the sacrificed mice for a biodistribution analysis. The animal study protocol was approved by the Ethics Committee of Guangxi Medical University Cancer Hospital (protocol code KY 2022-129/130 and approved on 25 February 2022) for studies involving animals. The tumor-bearing BALB/c mice were randomly allocated into four groups and injected with intravenous injections (*n* = 4): 1. Control; 2. NIR (0.8 W cm^−2^, 3 min); 3. PdRu@PEI (100 µgmL^−1^); 4. PdRu@PEI (100 µgmL^−1^) + NIR (3 min) at 1, 4, 7, 10, and 13 d. On days 1.5, 4.5, 7.5, 10.5, and 13.5, the 808 nm laser irradiation operation was executed. The body weights and tumor sizes were measured every 2 days. Finally, tumors and major organs of different groups were collected and sectioned for hematoxylin-eosin (H&E) staining.

### 2.12 Statistical analysis

All data from the experiments were directly applied for statistical analysis and presented as mean ± S. D. The probability (*p*) value was presented by different numbers of asterisks (*) according to its actual value (*p < 0.05; **p < 0.01; ***p < 0.001).

## 3 Results and discussion

### 3.1 Characterization analysis of PdRu@PEI nanoalloys

**FIGURE 1:**
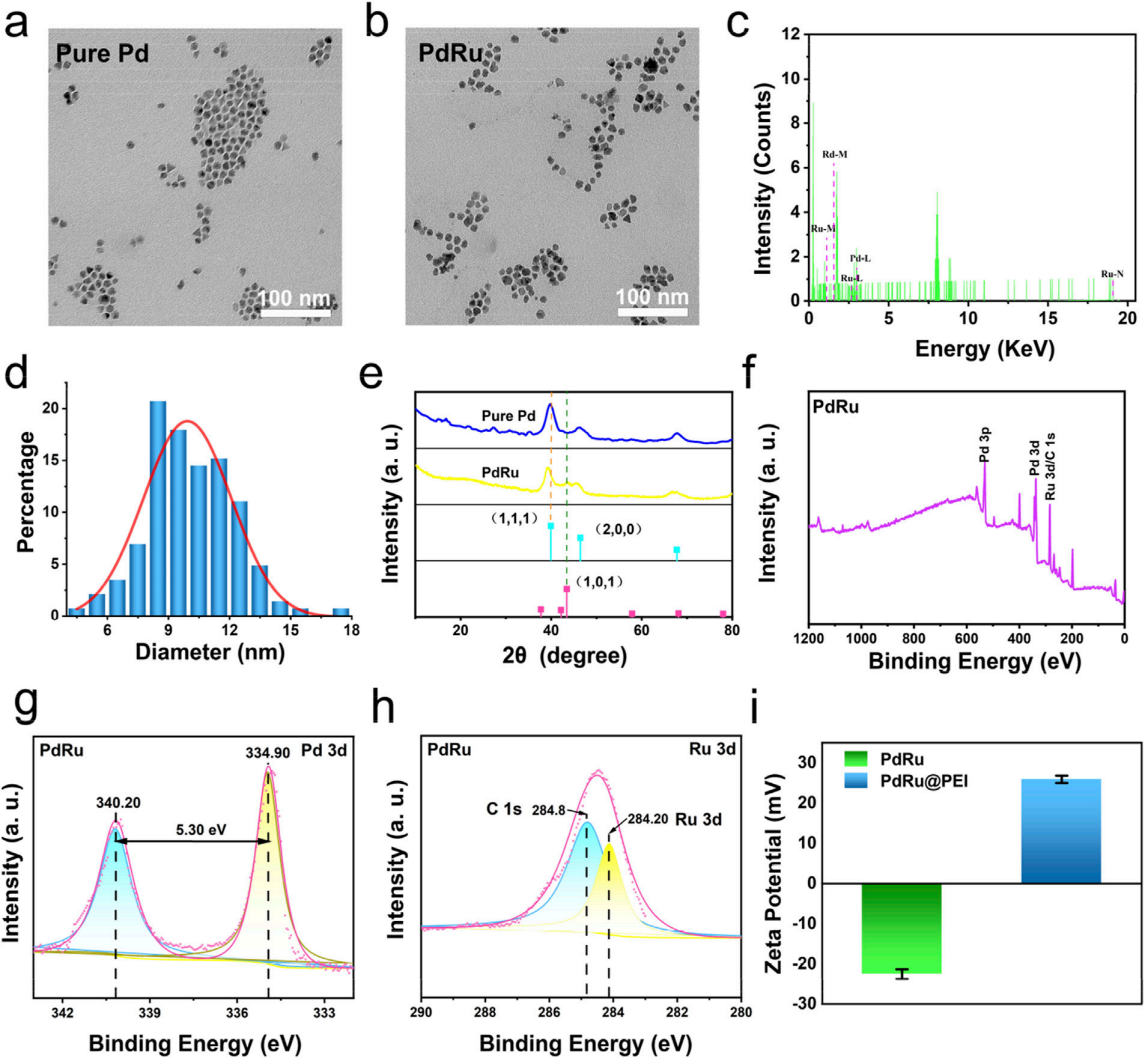
Characterization of PdRu@PEI nanoalloy. **(A)** TEM image of Pd nanoparticles. **(B)** TEM image of PdRu nanoalloy. **(C)** EDS energy spectrum of PdRu nanoalloy. **(D)** Size distribution of PdRu nanoalloy. **(E)** XRD patterns of Pd nanoparticles and PdRu nanoalloy. **(F)** Full XPS spectrum of PdRu nanoalloy. **(G)** High-resolution XPS spectrum of Pd 3d and **(H)** Ru 3d. **(I)** Zeta potentials of PdRu and PdRu@PEI nanoalloy.

The bimetallic nanoalloys PdRu@PEI were synthesized using RuCl_3_ and Pd (acac)_2_ as raw materials, with W(CO)_6_ as a reducing agent. Subsequently, PEI was applied to modify monodisperse PdRu@PEI nanoalloys for enhanced biocompatibility. TEM images depict the synthesized Pure Pd with an irregular morphology (Figure 1A; Supplementary Figure S1). In contrast, the nanoalloys exhibited a similar morphology but with the edges of the particles rougher (Figure 1B). The PdRu@PEI nanoalloys show a relatively uniform morphology while maintaining the smaller particle size characteristic of pure Pd (Supplementary Figure S2). Ru and Pd elements were identified in the energy-dispersive X-ray spectroscopy (EDS) spectrum of the PdRu nanoalloy, confirming successful preparation of PdRu nanoalloys (Figure 1C). Through statistics, the normal distribution trend and the fitted curve indicate an average particle size of 9.95 ± 0.21 nm for the PdRu nanoalloys (Figure 1D), with its ultra-small and relatively uniform morphology providing favorable conditions for subsequent experiments.

The XRD spectra of the PdRu bimetallic nanoalloys and pure Pd nanoparticles are presented in Figure 1E. Compared with pure Pd, the PdRu nanoalloys display a distinctive diffraction peak at 43.32°, consistent with the diffraction peak observed in the (101) lattice plane of the pure Ru standard spectrum. The disappearance of the characteristic (111) and (200) lattice planes, which belong to pure Pd, further confirms the successful preparation of the alloy. XPS analysis was performed on PdRu nanoalloys. XPS investigation spectrum shows that PdRu is composed of Pd and Ru composition (Figures 1F, H). The high-resolution XPS spectrum of Pd 3d shows two types of spin-orbits splitting, Pd 3d3/2 and Pd 3d5/2 (Figure 1G). The binding energies at 340.2 eV and 334.9 eV are attributed to Pd (0). Compared to pure Pd (citing senior sister paper data), the Pd 3d binding energy of PdRu nanoalloys decreased by 0.9 eV (Figure 1G). The binding energy confirms the formation of PdRu nanoalloy, and the electron transfer between Pd and Ru is speculated to exhibit excellent cooperation in POD-like enzyme activity. The Pd and Ru element concent were revealed by ICP-MS (Supplementary Table S1). To improve the biocompatibility of PdRu nanoalloys, we modified their surface with positively charged PEI (Figure 1I). The PdRu@PEI composite exhibits a positive charge of +25.93 mV, which is higher than the −22.50 mV recorded for the PdRu nanoalloy. This increase in positive charge signifies the successful functionalization with PEI, and such positive charges are beneficial for effectively targeting tumors through phagocytosis.

### 3.2 Nanozymes properties of PdRu@PEI nanoalloys

**FIGURE 2:**
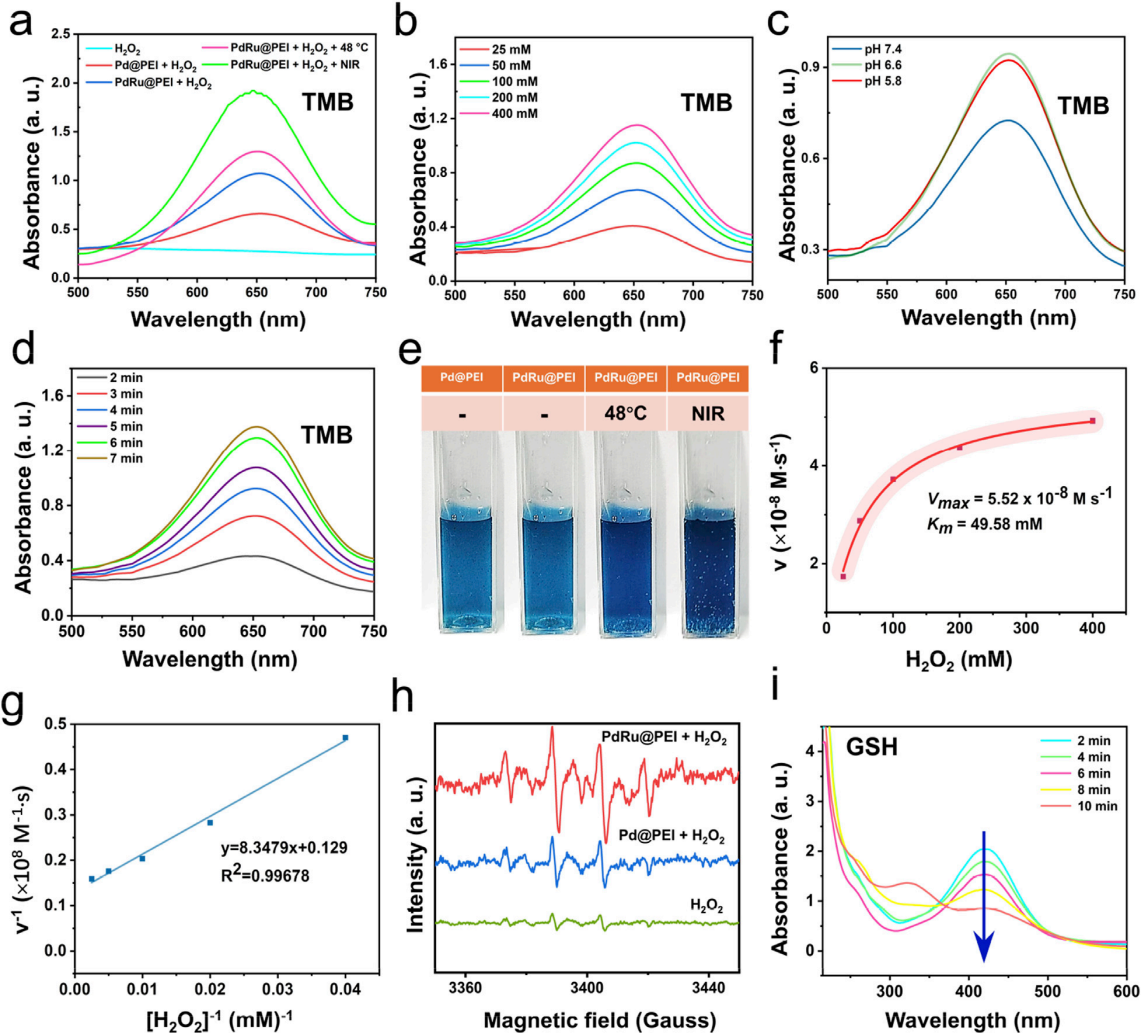
ROS production capacity of PdRu@PEI nanoalloy. **(A)** Oxidation of TMB to produce ·OH in different groups. **(B)** Material concentration-dependent oxidation of TMB indicates ·OH generation under various conditions. **(C)** Oxidation of TMB to produce ·OH in different pH conditions. **(D)** Time-dependent oxidation of TMB indicates ·OH generation. **(E)** Color response of TMB after being oxidized. **(F, G) **Enzyme kinetics spectrum under linear fitting, *K*_m_ and *V*_max_ of PdRu@PEI nanoalloys through regulated H_2_O_2_ concentration. **(H)** ESR spectra for Pd@PEI nanoparticles and PdRu@PEI nanoalloys in the presence of DMPO and H_2_O_2_. **(I)** Absorbance spectra of GSH depletion in 10 min.

Possible favorable conditions such as reduced Pd electron binding energy, amplified active sites, and mediation of electron transfer/valence state fluctuations enhance the POD-like activity in PdRu@PEI nanoalloys. After mixing PdRu@PEI with H_2_O_2_ solutions, H_2_O_2_ decomposed into ·OH in a time-dependentmanner, which could be monitored by a TMB substrate (Figure 2A; Supplementary Figure S3A). Compared with pure Pd, the absorbance of PdRu@PEI nanoalloys significantly improved, owing to amplified active sites and mediation of electron transfer/valence state fluctuations of bimetallic nanoalloys. ThePdRu@PEI nanoalloys, TMB, and H_2_O_2_ were placed in a 48°C water bath or excited by 808 nm laser irradiation, in which the absorbance at 652 nm both show significantly improved. Both temperatures increase and near-infrared light irradiation (NIR) can enhance the POD-like activity of PdRu@PEI nanoalloys. At the same time, the higher absorbance under 808 nm irradiation indicates the existent photodynamic potential of PdRu@PEI nanoalloy, which provided a complex therapeutic effect that exceeds photothermal therapy. As the concentration increased, the absorbance at 652 nm also increased (Figure 2B). Demonstrated by a positive correlation between concentration and reaction rate, the effective participation of PdRu@PEI as nanozymes in POD-like catalytic reactions has been reflected. At different pH values, the absorbance curve also varies, with the highest absorbance at a pH of 6.6 (Figure 2C), which shows the well potential usage of PdRu@PEI nanoalloys under the slightly acidic conditions of the TME. With time, the absorbance at 652 nm will gradually increase (Figure 2D), indicating that the process of PdRu@PEI anoalloys catalyzing the production of ·OH from H_2_O_2_ is stable and continuous. At the same time, photos were taken of the color reaction that occurred when TMB was oxidized to oxTMB in different states (Figure 2E), and the results were consistent with the UV-vis absorbance curve in Figure 2A. By adjusting the amount of the reaction substrate (H_2_O_2_), the relationship between substrate concentration and the reaction rate is obtained through the typical Michaelis−Menten dynamics (Figures 2F, G). Based on calculations of the corresponding Lineweaver-Burk plot, the maximum reaction rate (*V*_max_) and Michaelis-Menten constant (*K*_m_) of the PdRu@PEI nanoalloys were fitted to be 5.52 × 10^−8^ M s^−1^ and 49.58 mM, respectively, indicating the high affinity of nanoalloys for H_2_O_2_ substrates and the POD-like enzymatic activity. The production of ·OH was also verified by ESR measurement using a DMPO capture agent (Figure 2H), in which the typical 1:2:2:1 peak pattern confirms the presence of ·OH. Using DTNB as an indicator to observe the GSH content changes, the absorbance at 412 nm decreases with the extended incubation time (Figure 2I; Supplementary Figure S3B), confirming the ability of PdRu nanoalloys to possess a disrupted antioxidant system derived from glutathione peroxidase-like enzyme activity.

### 3.3 Photothermal effect of PdRu@PEI nanoalloys

**FIGURE 3:**
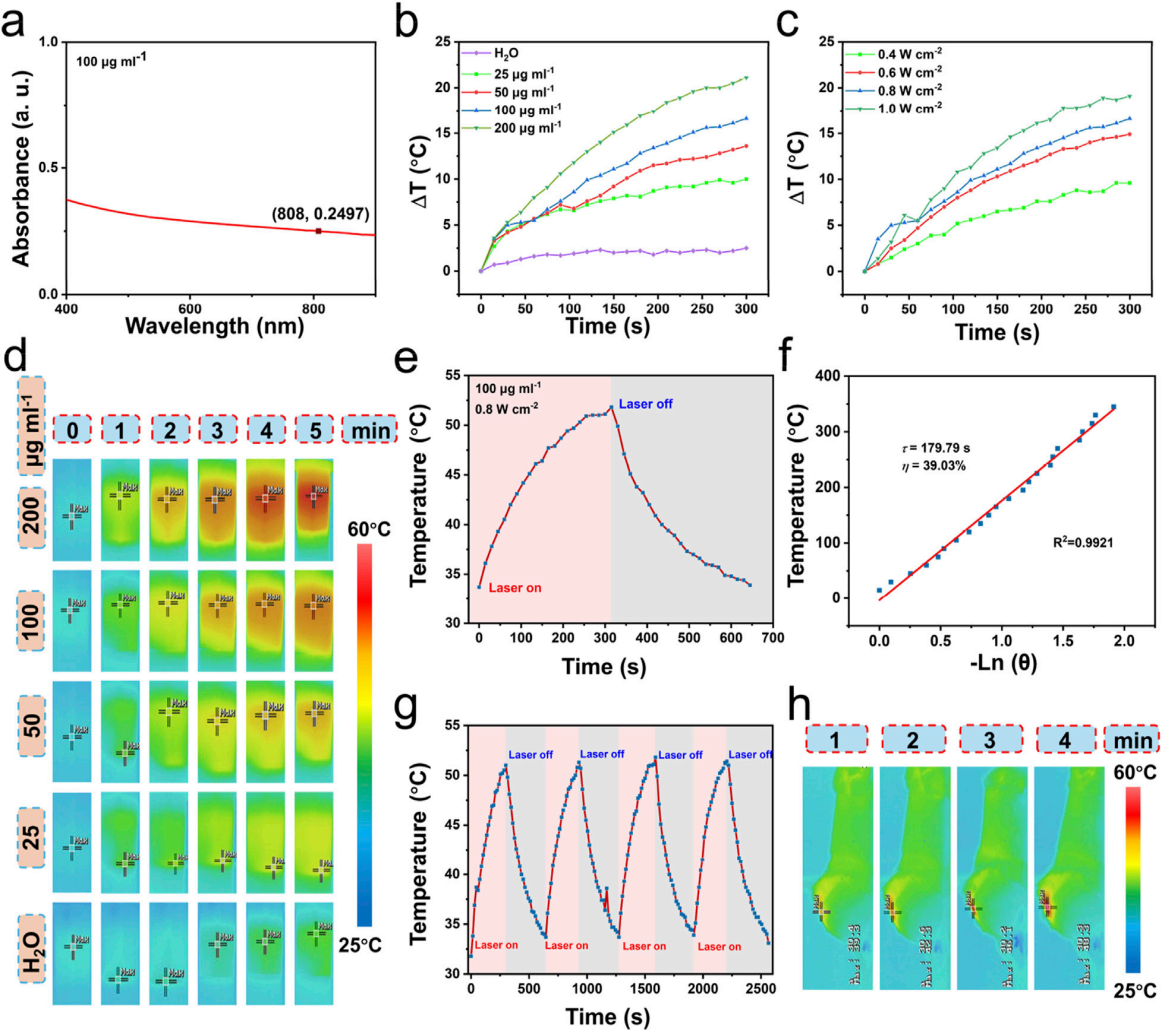
Photothermal properties of PdRu@PEI nanoalloys. **(A)** UV-vis absorption of PdRu@PEI nanoalloys. **(B)** Temperature-increasing curves of PdRu@PEI nanoalloys with different concentrations. **(C)** Temperature-increasing curves of PdRu@PEI nanoalloys under different concentrations under 808 nm NIR irradiation within 5 min. **(D)** Infrared thermal photos of the PdRu@PEI nanoalloys under 808 nm irradiation with different concentrations (0–5 min, photographed at 1 min intervals). **(E)** Heating and cooling temperature data of PdRu@PEI nanoalloy. **(F)** Fitting linear relationship of −Ln(*θ*)-T calculating from cooling data. **(G)** Recycling course of heating and cooling process. **(H)** *In vivo* photothermal effect of PdRu@PEI nanoalloy.

The PdRu@PEI nanoalloys exhibit good absorption intensity in the visible to near-infrared region (Figure 3A), indicating its potential as a photothermal agent. The 808 nm (0.8 W cm^−2^) laser irradiated PdRu@PEI nanoalloys solutions at different concentrations and recorded the results regularly. As shown in Figure 3B, when the laser irradiates the PdRu@PEI nanoalloy, even at low concentrations, the temperature will increase, and the change in pure water temperature after irradiation can be ignored. With the increase of PdRu@PEI nanoalloy concentrations, the heating rate increases due to its concentration-related manner. Recording the temperature changes in 5 min of irradiation at different laser powers with (0.4, 0.6, 0.8, and 1.0 W cm^−2^, 808 nm), the data increased from room temperature to 43.8, 47.3, 50.1, and 52.6°C, respectively (Figure 3C). In addition, we can visually observe the temperature rise differences of PdRu@PEI nanoalloys under different conditions from infrared thermography (Figure 3D). In order to further assess the photothermal performance of the alloy, the rising and falling temperatures of the PdRu@PEI nanoalloys (100 μg mL^−1^) were measured (Figure 3E). Using the data in the cooling process in Figure 3E as one of the photothermal parameters, the *τ*_s_ (179.79s) was calculated through the relationship between -Ln(*θ*) and temperature, and the photothermal conversion efficiency was determined as 39.03% (Figure 3F). The photothermal stability of PdRu@PEI nanoalloys is a key indicator for photothermal performance. Therefore, the PdRu@PEI nanoalloys were treated for four cycles of laser on/off process to revealthe photothermal stability (Figure 3G). The performance still maintains a similar rate of temperature increase and maximum temperature. The PdRu@PEI was injected into the mouse tumor *in situ*, and a significant warming phenomenon was observed under 808 nm laser irradiation (Figure 3H). With temperatures reaching 48.3°C after 4 min of irradiation, PdRu@PEI nanoalloys show good *in vivo* photothermal conversion ability.

### 3.4 *In vitro* anti-tumor evaluation of PdRu@PEI nanoalloys

**FIGURE 4:**
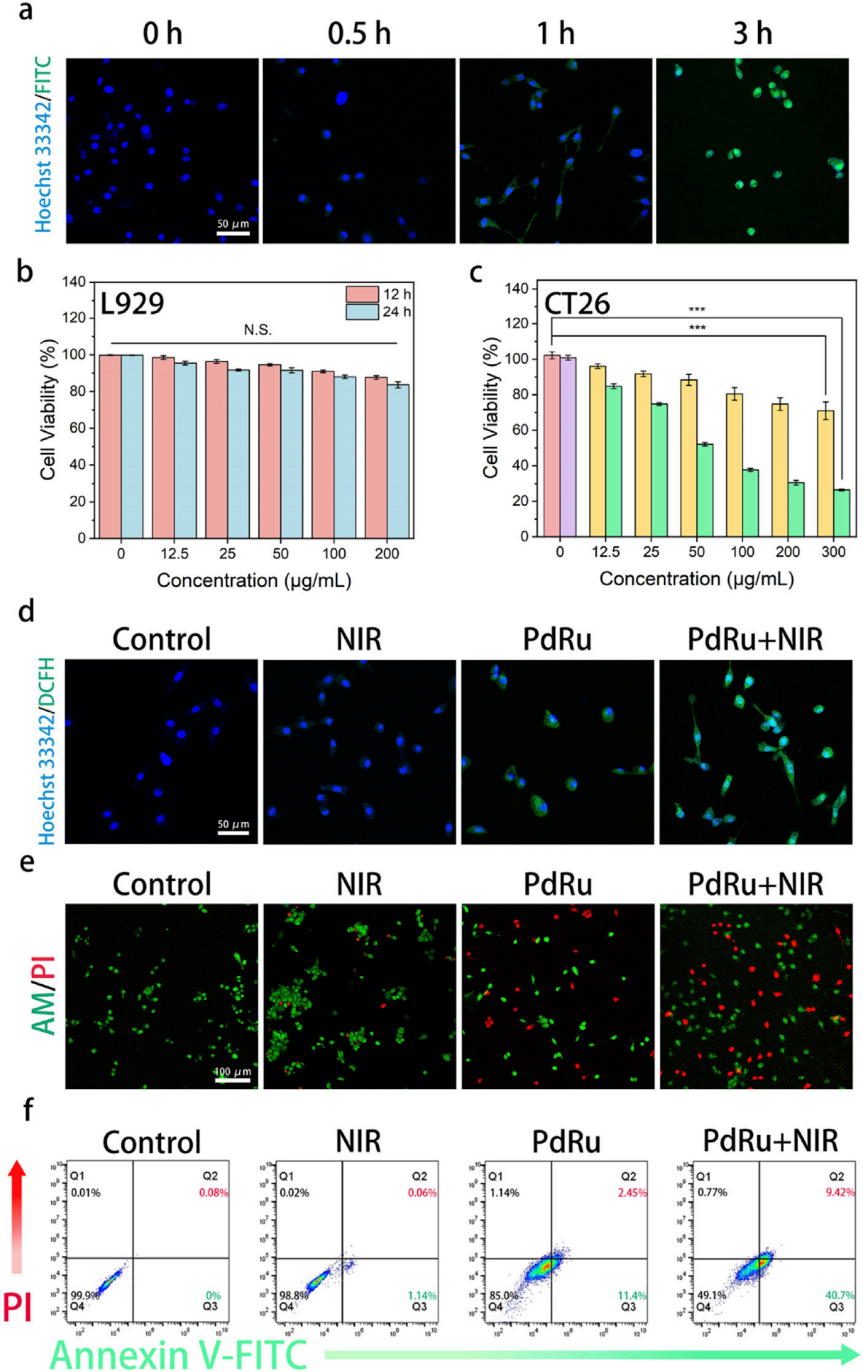
*In vitro* anti-tumor effect of PdRu@PEI nanoalloys. **(A)** The cellular uptake of FITC-labeled PdRu nanoalloys in CT26 cells. **(B, C)** Relative cell viabilities of L929 cells and cytotoxicity assay of CT26 cells. **(D)** ROS staining of CT26 cells. **(E)** AM/PI staining of CT26 cells. **(F)** Flow cytometry results.

To investigate the cellular internalization performance of PdRu@PEI, we labeled it with fluorescein isothiocyanate (FITC) and denoted it as FITC-labeled PdRu@PEI. The *in vitro* cellular uptake of FITC-labeled PdRu@PEI in CT26 cells was evaluated using CLSM. As shown in Figure 4A, the fluorescence signals of FITC were stronger with a prolonged incubation time, exhibiting that the cellular uptake of PdRu@PEI is time-dependent. As mentioned above, PdRu@PEI can be effectively taken up by tumor cells, achieving targeting function and more drug accumulation at tumor sites. The cytocompatibility was evaluated using a methyl thiazolyl tetrazolium (MTT) assay on the L929 cell line (Figure 4B). The experiment was repeated 4 times (*n* = 4). After endocytosis of the PdRu@PEI nanoalloy, L929 cells were incubated at different times (12 h and 24 h). Through the cell viabilities revealed in Figure 4B, PdRu@PEI nanoalloys are compatible with healthy cells and did not cause excessive cell death. The cytotoxicity of PdRu@PEI nanoalloys was evaluated using an MTT assay on CT26 cells (Figure 4C), repeated 4 times (*n* = 4). The PdRu@PEI exhibits concentration-dependent cytotoxicity in CT26 cells. The difference in mortality is attributed to the characteristics of L929 cells, which differ from CT26 cells in lacking an acidic environment and high levels of H_2_O_2_ to generate sufficient ROS for cytotoxicity. Cytotoxicity to CT26 cells was further enhanced when irradiated by an 808 nm laser, as the photothermal effect enhances the synergistic effect of POD-like activity and oxidative stress. The degree of intracellular oxidative stress was measured to analyze ROS production after co-culturing with different samples using the DCFH-DA probe (Figure 4D). Compared with the other groups, the green fluorescence level in PdRu@PEI + NIR group was significantly increased, which indicated that the combination of NIR and PdRu@PEI could achieve higher ROS production. Living and dead cells can be stained with Calcein-AM/PI assay to visualize the anticancer efficacy with CLSM (Figure 4E). The trends of cell apoptosis under different treatments can be tested by annexin V-FITC/PI through flow cytometry assay (Figure 4F). Consistent with the intracellular ROS assay results, the control or NIR groups rarely cause cell apoptosis. The PdRu@PEI group showed partial apoptosis due to the enzyme-like activity of the Pd and Ru. As expected, the CT26 cells treated with PdRu@PEI + NIR showed the most apoptosis, providing strong evidence that photothermal-strengthened POD activity and oxidative stress co-enhanced ROS generation, ultimately leading to apoptosis of a significant number of tumor cells.

### 3.5 *In vivo* anti-tumor evaluation of PdRu@PEI nanoalloys

**FIGURE 5:**
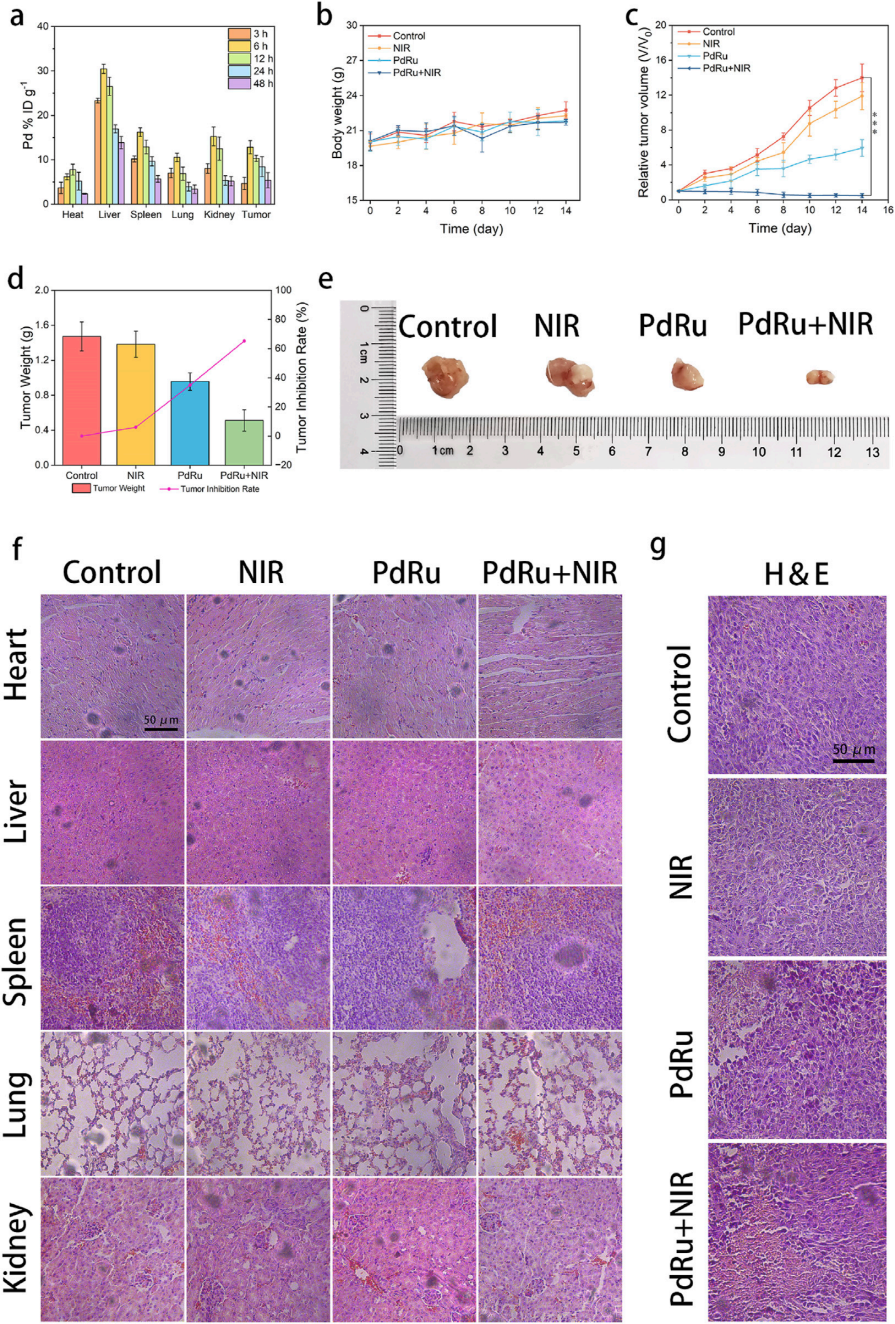
*In vivo* anti-tumor effect of PdRu@PEI nanoalloys. **(A)** Biodistribution of PdRu@PEI nanoalloys in tumors and main organs. **(B)** Body weight and **(C)** relative tumor volume changes of tumor in the mice. **(D)** Tumor weight in different groups. **(E)** Representative tumor photos in different groups. **(F)** H&E-staining of main organ slices in various groups. **(G)** H&E-staining of tumor slices in various groups.

Additionally, we investigated the *in vivo* anti-tumor ability of PdRu@PEI nanozymes. First, the distribution of PdRu@PEI nanoalloy was evaluated. The mice were executed after injection (3, 6, 12, 24, and 48 h), and the Pd ions content was obtained by ICP-MS for the biodistribution investigation of PdRu@PEI in the major organs and tumor tissues (Figure 5A). Compared to other organs, the increased biodistribution in the liver, spleen, and kidney might be attributed to endothelium reticulum system clearance. The increase in Pd ions content in tumor tissues within 6 h demonstrates superior targeting properties.

We then studied its tumor-suppressive ability in tumor-bearing animals. The BALB/c mice were purchased and harbored CT26 tumors. The tumor-bearing BALB/c mice were randomly allocated into four groups (groups were set as Control, NIR, PdRu@PEI, and PdRu@PEI + NIR, *n* = 4) and injected with intravenous injections at 1, 4, 7, 10, and 13 days. The average weights of mice in each group were similar throughout the treatment period (Figure 5B). Track of average tumor growth was tested through calculated volumes (*V*/*V*_*0*_, *V* = 1/2*w* ^2^ *l*, *V*_*0*_ = original average volume on day 0) (Figure 5C). The comparable growth of relative tumor volumes between the control and the NIR groups reveals the marginal slowed tumor development brought by pure NIR irradiation. The PdRu@PEI group inhibited tumor development, demonstrating that its enzyme activity may destroy tumors, however the impact was limited. The PdRu@PEI + NIR group had the strongest tumor inhibitory effect, with the tumor virtually totally suppressed due to NIR-mediated enzymatic activity. Following treatment completion, the subcutaneous tumors were collected and weighed to determine the rate of tumor suppression. Tumor suppression rates were subsequently estimated using tumor weight (Figure 5D). The PdRu@PEI + NIR group demonstrated a maximal inhibition ratio of 65.4%, much higher than the other groups. The results demonstrated that combining photothermal effect-enhanced POD-like activity of PdRu nanoalloys had great *in vivo* anti-tumor effects. The example photos of representative tumors in various groups supported these findings (Figure 5E). After the therapy, the primary organs and tumor sections were histologically examined. The major organs suffered no substantial harm after treatments (Figure 5F). The H&E-staining experiment revealed that the tumor tissues in the PdRu@PEI group were badly damaged, but the controls and NIR groups showed no significant alterations. Similarly, the most severe damage occurred following the PdRu@PEI + NIR treatment (Figure 5G). These results confirm that PdRu@PEI promotes apoptosis *in vivo* and can be used as an innovative nanoplatform for tumor therapy.

## 4 Conclusion

The PdRu@PEI bimetallic nanoalloys were synthesized using the co-reduction approach to provide a tumor therapy platform combining improved photothermal effect and enhanced POD-like activity. *In vitro* and *in vivo* anti-tumor tests demonstrated that the PdRu@PEI bimetallic nanoalloys have POD-like activity. The GSH depletion ability provided by PdRu@PEI can restore POD-like enzymatic activity. Furthermore, under 808 nm laser irradiation, the PdRu@PEI bimetallic nanoalloys superior light absorption characteristics and photothermal conversion efficiency were enhanced to boost their photothermal capabilities via the photothermal effect. The locally higher temperature further boosted PdRu@PEI bimetallic nanoalloys catalytic production of ROS. Furthermore, the nanoalloys caused oxidative stress by raising ROS levels, continually depleting GSH, and affecting antioxidant system homeostasis. Overall, PdRu@PEI, a trinity of POD-like activity, photothermal effect, and oxidative stress, demonstrated excellent anti-tumor efficacy both *in vitro* and *in vivo*.

## Data Availability

The original contributions presented in the study are included in the article/Supplementary Material, further inquiries can be directed to the corresponding authors.

## References

[B1] Ai Y. He M.-Q. Sun H. Jia X. Wu L. Zhang X. (2023). Ultra-small high-entropy alloy nanoparticles: efficient nanozyme for enhancing tumor photothermal therapy. Adv. Mater. 35, 2302335. 10.1002/adma.202302335 36995655

[B2] Chen S. Li B. Yue Y. Li Z. Qiao L. Qi G. (2024). Smart nanoassembly enabling activatable NIR fluorescence and ROS generation with enhanced tumor penetration for imaging‐guided photodynamic therapy. Adv. Mater. 36, e2404296. 10.1002/adma.202404296 38685574

[B3] Diao L. Liu M. (2023). Rethinking antigen source: cancer vaccines based on whole tumor cell/tissue lysate or whole tumor cell. Adv. Sci. (Weinh) 10, e2300121. 10.1002/advs.202300121 37254712 PMC10401146

[B4] Duan F. Jia Q. Liang G. Wang M. Zhu L. McHugh K. J. (2023). Schottky junction nanozyme based on Mn-bridged Co-phthalocyanines and Ti_3_C_2_T_x_ nanosheets boosts integrative type I and II photosensitization for multimodal cancer therapy. ACS Nano 17 (12), 11290–11308. 10.1021/acsnano.2c12270 37276377

[B5] Huang Y. Ren J. Qu X. (2019). Nanozymes: classification, catalytic mechanisms, activity regulation, and applications. Chem. Rev. 119 (6), 4357–4412. 10.1021/acs.chemrev.8b00672 30801188

[B6] Jiang W. Zhong S. Chen Z. Qian J. Huang X. Zhang H. (2023). 2D-CuPd nanozyme overcome tamoxifen resistance in breast cancer by regulating the PI3K/AKT/mTOR pathway. Biomaterials 294, 121986. 10.1016/j.biomaterials.2022.121986 36623325

[B7] Liao H. Yang S. Liang Z. Xiao L. Xie S. Lin P. (2023). A cancer cell selective replication stress nano amplifier promotes replication fork catastrophe to overcome radioresistance. ACS Nano 17, 18548–18561. 10.1021/acsnano.3c06774 37706454

[B8] Nie D. Ling Y. Lv W. Liu Q. Deng S. Shi J. (2023). *In situ* attached photothermal immunomodulation-enhanced nanozyme for the inhibition of postoperative malignant glioma recurrence. ACS Nano 17, 13885–13902. 10.1021/acsnano.3c03696 37399132

[B9] Pan Q. Lin F. Liu R. Li Y. Zhang X. Luo R. (2023). Fe/Ni layered double hydroxide biocatalysts inhibit tumor growth through ROS and ferroptosis signaling pathway. Chem. Eng. J., 466. 10.1016/j.cej.2023.142962

[B10] Rong M. Liu J. Sun Z. Li T. Li Y. Jiang C. (2023). Rational utilization of black phosphorus nanosheets to enhance palladium‐mediated bioorthogonal catalytic activity for activation of therapeutics. Angew. Chem. Int. Ed. 62 (19), e202216822. 10.1002/anie.202216822 36917027

[B11] Shi X. Shu L. Wang M. Yao J. Yao Q. Bian S. (2023). Triple-combination immunogenic nanovesicles reshape the tumor microenvironment to potentiate chemo-immunotherapy in preclinical cancer models. Adv. Sci. (Weinh) 10 (15), e2204890. 10.1002/advs.202204890 37017572 PMC10214259

[B12] Song C. Zhang X. Gao Z. Wei Z. Zhou M. Wang Y. (2023). Regulating tumor cholesterol microenvironment to enhance photoimmunotherapy in oral squamous cell carcinoma. Chem. Eng. J., 462. 10.1016/j.cej.2023.142160

[B13] Tao J. Tian Y. Chen D. Lu W. Chen K. Xu C. (2023). Stiffness-transformable nanoplatforms responsive to the tumor microenvironment for enhanced tumor therapeutic efficacy. Angew. Chem. Int. Ed. Engl. 62 (7), e202216361. 10.1002/anie.202216361 36524465

[B14] Wang D. Zhu X. Wang X. Wang Q. Yan K. Zeng G. (2023d). Protocells capable of generating a cytoskeleton-like structure from intracellular membrane-active artificial organelles. Adv. Funct. Mater. 33, 2306904. 10.1002/adfm.202306904 38344241 PMC10857770

[B15] Wang H. Gao Z. Jiao D. Zhang Y. Zhang J. Wang T. (2023a). A microenvironment dual‐responsive nano‐drug equipped with PD‐L1 blocking peptide triggers immunogenic pyroptosis for prostate cancer self‐synergistic immunotherapy. Adv. Funct. Mater. 33 (16). 10.1002/adfm.202214499

[B16] Wang R. Qiu M. Zhang L. Sui M. Xiao L. Yu Q. (2023c). Augmenting immunotherapy via bioinspired MOF‐based ROS homeostasis disruptor with nanozyme‐cascade reaction. Adv. Mater. 35, e2306748. 10.1002/adma.202306748 37689996

[B17] Wang S. Huang J. Zhu H. Zhu J. Wang Z. Xing Y. (2023b). Nanomodulators capable of timely scavenging ROS for inflammation and prognosis control following photothermal tumor therapy. Adv. Funct. Mater. 33 (21). 10.1002/adfm.202213151

[B18] Wang S. Zhao J. Zhang L. Zhang C. Qiu Z. Zhao S. (2021). A unique multifunctional nanoenzyme tailored for triggering tumor microenvironment activated NIR-II photoacoustic imaging and chemodynamic/photothermal combined therapy. Adv. Healthc. Mater 11, e2102073. 10.1002/adhm.202102073 34731532

[B19] Wen D. Li K. Deng R. Feng J. Zhang H. (2023). Defect-rich glassy IrTe2 with dual enzyme-mimic activities for sono-photosynergistic-enhanced oncotherapy. J. Am. Chem. Soc. 145 (7), 3952–3960. 10.1021/jacs.2c09967 36757875

[B20] Wu M. Xue L. Guo Y. Dong X. Chen Z. Wei S. (2023). Microenvironmentally responsive chemotherapeutic prodrugs and CHEK2 inhibitors self-assembled micelles: protecting fertility and enhancing chemotherapy. Adv. Mater 35 (11), e2210017. 10.1002/adma.202210017 36528787

[B21] Xiaoli C. Lei J. Hongye Y. Wu Y. Gu W. Du D. (2021). Nanozyme-involved biomimetic cascade catalysis for biomedical applications. Mater. Today 44, 211–228. 10.1016/j.mattod.2020.12.005

[B22] Xu Z. Luo Q. He Y. He Y. Zhang X. Wang J. (2024). Endogenous nitric oxide releases *in situ* for RNS/ROS synergistic cancer therapy. Adv. Funct. Mater. 34. 10.1002/adfm.202314536

[B23] Yang L. Wang D. Jia H. Yang C. Zhang Y. Li H. (2023c). Tumor‐specific peroxynitrite overproduction disrupts metabolic homeostasis for sensitizing melanoma immunotherapy. Adv. Mater. 35 (29), e2301455. 10.1002/adma.202301455 37133969

[B25] Yang X. Xu C. Zhang X. Li P. Sun F. Liu X. (2023b). Development of sulfonamide‐functionalized charge‐reversal AIE photosensitizers for precise photodynamic therapy in the acidic tumor microenvironment. Adv. Funct. Mater. 33 (30). 10.1002/adfm.202300746

[B26] Yang Y. Hu T. Bian Y. Meng F. Yu S. Li H. (2023a). Coupling probiotics with 2D CoCuMo-ldh nanosheets as a tumor-microenvironment-responsive platform for precise NIR-II photodynamic therapy. Adv. Mater 35 (23), e2211205. 10.1002/adma.202211205 36913539

[B27] Yu F. Shange L. Yu Y. Rong H. Zhang J. (2021). Catalytic nanomaterials toward atomic levels for biomedical applications: from metal clusters to single-atom catalysts. ACS Nano 15, 2005–2037. 10.1021/acsnano.0c06962 33566564

[B28] Zandieh M. Liu J. (2021). Nanozyme catalytic turnover and self-limited reactions. ACS Nano 15, 15645–15655. 10.1021/acsnano.1c07520 34623130

[B29] Zeng F. Fan Z. Li S. Li L. Sun T. Qiu Y. (2023). Tumor microenvironment activated photoacoustic-fluorescence bimodal nanoprobe for precise chemo-immunotherapy and immune response tracing of glioblastoma. ACS NANO 17, 19753–19766. 10.1021/acsnano.3c03378 37812513

[B30] Zhang H. Cui M. Tang D. Wang B. Liang G. Xu C. (2024c). Localization of cancer cells for subsequent robust photodynamic therapy by ROS responsive polymeric nanoparticles with anti‐metastasis complexes NAMI‐A. Adv. Mater. 36, e2310298. 10.1002/adma.202310298 38145801

[B31] Zhang Q. Luo Q. Liu Z. Sun M. Dong X. (2023b). Nano-ROS-generating approaches to cancer dynamic therapy: lessons from nanoparticles. Chem. Eng. J., 457. 10.1016/j.cej.2022.141225

[B32] Zhang R. Jiang B. Fan K. Gao L. Yan X. (2024b). Designing nanozymes for *in vivo* applications. Nat. Rev. Bioeng. 2, 849–868. 10.1038/s44222-024-00205-1

[B33] Zhang W. Wang M. Liu B. Chen H. Tan J. Meng Q. (2024a). Glutathione induced *in situ* synthesis of Cu single‐atom nanozymes with anaerobic glycolysis metabolism interference for boosting cuproptosis. Angew. Chem. Int. Ed. 63, e202402397. 10.1002/anie.202402397 38389036

[B34] Zhang W. Wang M. Liu B. Yuan M. Yang Z. Tan J. (2023a). Rational design of Multi-model ROS regulation Nano-platform for enhanced Mild-temperature photothermal therapy. Chem. Eng. J., 460. 10.1016/j.cej.2023.141818

[B35] Zhao P. Li H. Bu W. (2023a). A forward vision for chemodynamic therapy: issues and opportunities. Angew. Chem. Int. Ed. 62 (7), e202210415. 10.1002/anie.202210415 36650984

[B36] Zhao Q. Zheng L. Gao Y. Li J. Wei J. Zhang M. (2023b). Dual active centers linked by a reversible electron station as a multifunctional nanozyme to induce synergetically enhanced cascade catalysis for tumor-specific therapy. J. Am. Chem. Soc. 145 (23), 12586–12600. 10.1021/jacs.3c01532 37277963

[B38] Zhou Y. Yuan J. Xu K. Liu Y. (2024). Nanotechnology reprogramming metabolism for enhanced tumor immunotherapy. ACS Nano 18 (3), 1846–1864. 10.1021/acsnano.3c11260 38180952

[B39] Zhu P. Pu Y. Wang M. Wu W. Qin H. Shi J. (2023a). MnOOH-catalyzed autoxidation of glutathione for reactive oxygen species production and nanocatalytic tumor innate immunotherapy. J. Am. Chem. Soc. 145 (10), 5803–5815. 10.1021/jacs.2c12942 36848658

[B40] Zhu P. Zhou C. Chen J. Chu Q. Li F. Fu Y. (2023b). Propionibacterium acnes cloaked with ZnAl layered double hydroxides synergistically inhibits tumor growth and metastasis. Adv. Funct. Mater. 33 (25). 10.1002/adfm.202214105

